# Gait Pattern Differences Between Young Adults and Physically Active Older Adults

**DOI:** 10.3390/medicina61101752

**Published:** 2025-09-25

**Authors:** Carmen García-Gomariz, Fernando Domínguez-Navarro, Mercedes María Fernández-Benet, José-María Blasco, David Hernández-Guillén, Enrique Sanchis-Sales

**Affiliations:** 1 Department of Nursing, University of Valencia, Menéndez y Pelayo Av. 19, 46010 Valencia, Spain; carmen.garcia-gomariz@uv.es (C.G.-G.); ferbenet94@gmail.com (M.M.F.-B.); enrique.sanchis-sales@uv.es (E.S.-S.); 2 Group of Research Advances in Ankle and Foot, Department of Nursing, University of Valencia, 46010 Valencia, Spain; 3 Group of Physiotherapy in the Aging Process: Social and Health Care Strategies, University of Valencia, 46010 Valencia, Spain; jose.maria.blasco@uv.es (J.-M.B.); david.hernandez@uv.es (D.H.-G.); 4 Department of Physiotherapy, University of Valencia, Calle Gascó Oliag 5, 46010 Valencia, Spain

**Keywords:** gait, biomechanics, older adult, physical activity

## Abstract

*Background and Objectives*: This study aimed to compare gait patterns between young adults and physically active older adults. Additionally, the relation between these parameters and age was explored. *Materials and Methods*: Transversal case and control study, recruiting 81 participants divided into two groups: young adults (18–45 years) and physically active older adults (60+ years). Participants were assessed using the PodoSmart Insole^®^ system, which recorded spatiotemporal and kinematic gait data. Gait parameters were measured during a self-selected walking test. Data analysis included descriptive statistics, *t*-tests for group comparisons, and Pearson’s correlation to explore relationships between age and gait parameters. *Results*: Significant differences in gait parameters were found between young and older adults, particularly in stride length (right foot: *p* = 0.009, left foot: *p* = 0.001), cadence (*p* < 0.001), contact time (*p* < 0.001), swing time (*p* < 0.001), and support phase duration (*p* < 0.001), with moderate to large effect sizes. Sex differences were also observed within each group for several gait variables. Correlation analysis evidenced worsened parameters with increasing age, with moderate to strong associations in terms of cadence (r = −0.590), contact time (r = −0.504, r = −0.462), swing time (r = −0.662), and support phase duration (r = −0.524, r = −0.439). *Conclusions*: Evident differences in gait parameters are observed between young adults and active older adults. Although these results follow the trend of previous studies that employed more sophisticated lab-based protocols for gait analysis, slight differences between our study and these others could be attributed to the regular physical activity performed by these participants, which should be explored in more detail in future studies.

## 1. Introduction

Aging entails a gradual and person-specific decline in cognitive and physical capacities [1]. Among the most affected systems is the musculoskeletal, which undergoes a cascade of degenerative changes that adversely impact locomotion, including proprioceptive and neuromuscular alterations, joint stiffness, limited mobility, and muscle atrophy—common age-related factors that contribute to a decline in walking ability [2,3]. However, significant variability exists in walking capacity within similar age groups, with regular physical exercise habits playing a major role in the overall performance [4,5].

Due to the critical role of walking in daily physical activities [6] and the strong association with quality of life [7], gait analysis is of paramount relevance to capture the overall health status of individuals during aging. Consequently, various biomechanical studies have been conducted using kinematic, dynamometric, or infrared measurement systems to analyze gait patterns in older adults [8,9,10]. These studies have revealed a general trend for old people to adjust gait parameters in ways that compensate for reduced balance ability, albeit with diminished efficiency. Concretely, gait speed has been observed to markedly decrease with every passing decade [11]. Additionally, stride length and width, as well as the duration of double support, are adjustments commonly observed in older adults to promote greater stability [12,13].

Although these procedures are considered the gold standard in laboratories or well-equipped hospitals due to their high precision and clinical validation [14], their elevated economic cost and the need for technically skilled personnel limit their implementation in all clinical settings.

As an alternative to these costly devices, wearable inertial motion capture systems, which can be easily integrated into shoes or clothing, are gaining interest due to their lower cost and user-friendly design, making them more accessible for clinical applications [15]. Indeed, one example is PodoSmart Insoles^®^, a portable and lightweight system that enables collecting gait parameters during different daily activities, including walking and running [16]. It connects to a smart device via Bluetooth and uses artificial intelligence to calculate gait variables such as speed, stride length, and double support time. This system has been validated and has shown excellent reliability [17,18].

Given the continuous effort to assess the health status of older adults through gait analysis, employing more affordable and easy-to-use technology may help both researchers and clinicians collect useful data applicable to health management in this population [19].

Although the effects of aging on walking parameters and the impact of regular activity are well documented, it remains unclear whether, and to what extent, a portable and innovative sensor system can detect gait differences related to aging and regular activity. Therefore, the present study aimed to compare gait patterns between young adults and physically active older adults, as well as to analyze the influence of sex.

## 2. Materials and Methods

### 2.1. Design and Ethics

In this observational study, a biomechanical analysis of the kinematic parameters of gait was conducted in adults of two age ranges. Gait parameters were assessed with the PodoSmart Insoles^®^ system [18]. The sample was recruited from May to July 2023 in the Don Rafael Romeu primary health-care center and the municipal sports facilities of Enguera (Valencia, Spain). The assessments were conducted in the Podiatric Clinic of the Universitat de Valencia (Fundació Lluís Alcanyís, Valencia, Spain). The study design adhered to the scientific and ethical principles set in Helsinki, and the procedures were approved by the Ethical Board of the Universitat de València (Spain) (nº 2528046/2023). All participants were informed verbally and in writing about the study and signed a consent form to participate.

### 2.2. Participants

The study included two groups of participants. The first group consisted of young adults aged between 18 and 45 years. Inclusion was based on participant screening to confirm that individuals did not have a known musculoskeletal condition that could affect their normal gait function, nor any traumatic injury or operation in the last 6 months, such as a sprain or prosthesis.

The second group consisted of physically active adults over 60 years old. To meet this requirement, the participants were recruited from the Enguera Sé Saludable program, a nationally funded project that enrolls people over 60 from the rural municipality of Enguera (Spain) to practice 2 weekly sessions of multimodal training (1 h per day) and another weekly day of walking 10 km. Individuals were excluded from the study if they used walking aids, had prostheses or orthoses, or suffered from osteoarticular, muscular, neurological, cognitive or rheumatic conditions that impaired their ability to engage in an active lifestyle. Additionally, participants with feet exhibiting high pronation or supination, as assessed using the Foot Posture Index (FPI), were also excluded.

### 2.3. Procedures

The principal investigator of this research was in charge of screening the sample, verifying compliance with the inclusion criteria, and collecting informed consent. A podiatrist, with more than ten years of experience, assessed all participants, regardless of group. The principal investigator tabulated the measurements in an Excel spreadsheet and anonymized the database with numerical codes for each participant. The biometrician of this research analyzed the anonymized raw data.

### 2.4. Measures

Demographic and anthropometric data of participants, such as sex, age, height, weight, and shoe size, were collected. Gait parameters were assessed using the PodoSmart Insoles^®^ (Digitsole SAS, Nancy, France), a wearable system designed to measure gait variables in real-life conditions. The system consists of wireless sensors integrated in a sole that can be fitted into any shoe, providing spatial, temporal, and kinematic gait data. Artificial intelligence is employed to collect and analyze the data acquired from the sensors during walking, transforming it into a user-friendly format displayed on a mobile app.

All measurements were conducted in the morning (from 9:00 to 11:00 a.m.), with participants asked to attend within this time frame during the study period. No specific instructions were given regarding the order of assessments, which was adapted according to the participants’ preferences and availability. For the analysis, each participant was instructed to walk at a self-selected, comfortable speed for 2 min along an 18 m corridor. The insole was placed in the shoe following the previously described protocol. Participants were asked to wear their regularly used shoes to ensure comfort and to avoid abnormal gait patterns. Before formal testing, a verbal explanation and demonstration were provided by the researcher. Participants then performed the test twice, and the average of each parameter was calculated. To ensure robust and feasible results, only the walking parameters that have been previously validated and demonstrated good–excellent reliability were used for analysis [16,18]. Hence, from all the gait parameters derived from the PodoSmart system, those selected to analyze were: stride length, cadence, speed, contact time, swing time, double support, support phase duration, heel strike angle, toe-off progression angle, propulsion phase duration, step progression angle, stepping, circumduction. The description of these variables and the ICC values reported previously [16,18] are shown in Table 1. ICC was catalogued as follows: minor reliability <0.5; moderate reliability 0.51 to 0.75; high reliability 0.76 to 0.9; excellent reliability >0.9.

### 2.5. Data Analysis

A descriptive statistical analysis was conducted to synthesize the characteristics of the sample using measures of central tendency (mean, minimum, maximum, etc.), measures of asymmetry, and perform dispersion tests (standard deviation). Normality of the data was assessed for each group independently, using the Kolmogorov–Smirnov test to determine whether the evaluated parameters followed a normal distribution. For the comparison of gait parameters between total sample of young and physically active older adults, independent sample *t*-tests were performed. Effect sizes were calculated as Cohen’s d using pooled standard deviation. Descriptors were applied as follows: small 0.2 ≤ |d| < 0.5, medium 0.5 ≤ |d| < 0.8, large |d| ≥ 0.8. Additionally, specific values according to sex (men vs. women) were also analyzed, both within the same group and across different groups. To explore associations between gait parameters and age, Pearson’s correlation analysis was conducted. The strength of correlation was interpreted as follows: weak (r = 0.1–0.3), moderate (r = 0.3–0.5), and strong (r > 0.5). The SPSS software (IBM, Armonk, NY, USA) version 25.0 was used to perform the analyses. Significant values were set at *p* < 0.05.

## 3. Results

**Figure 1:**
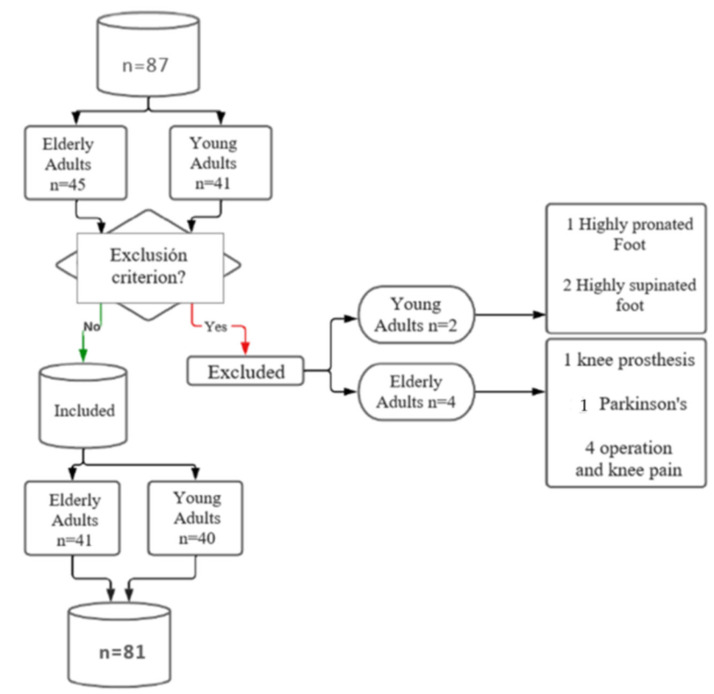
Flow chart of participants.

A total of 87 individuals were initially screened. After applying the inclusion criteria, 81 participants were eligible and included in the analysis, comprising two groups: young adults (*n* = 40, female = 50%) and physically active older adults (*n* = 41, female = 75.6%). Reasons for exclusion included the use of a prosthesis that interfered with normal walking (*n* = 2), a highly pronated foot (*n* = 2), Parkinson’s disease (*n* = 1), and a recent knee operation (*n* = 1). A flowchart of participant selection is shown in Figure 1.

Demographic and anthropometric characteristics of each group are exposed in Table 2, revealing obvious significant differences in age, and in terms of weight, BMI, and Foot Posture Index. The results of the *t*-test revealed significant differences in several spatiotemporal parameters among the two age groups. Concretely, these differences were observed for stride length (right foot: *p* = 0.009, left foot: *p* = 0.001), cadence (*p* < 0.001), contact time (right and left foot *p* < 0.001), swing time (right and left foot *p* < 0.001), support phase (right and left foot *p* < 0.001), toe-off progression angle (right foot: *p* = 0.042, left foot: *p* = 0.012), and left foot step progression angle (*p* = 0.012). The effect sizes were predominantly medium to large among significant outcomes. Notably, large effects were found for swing time (right: d = 1.85; left: d = 1.81) and support phase time (right: d = 1.16; left: d = 0.92). (Table 3). Additionally, sex was found to be relevant for certain parameters, as significant differences were observed within each age group for several variables, as shown in Table 4.

Correlation analysis further confirmed the influence of age on gait function, with significantly moderate to strong correlations observed for cadence (r = −0.590), contact time (right foot: r = −0.504, left foot: r = −0.462), swing time (right foot: r = −0.662, left foot: r = −0.660), and support phase duration (right foot: r = −0.524, left foot: r = −0.439). Detailed correlation results are exposed in Table 5.

## 4. Discussion

This study aimed to compare gait patterns between young adults and physically active older adults using the novel and easy-to-implement PodoSmart Insole^®^ system, as well as to analyze the influence of sex and explore the impact of aging on these outcomes. The results obtained follow the trend of previous studies, indicating a reduced efficacy in gait parameters with increasing age. Specifically, active older adults exhibited lower step length with increased cadence, a shorter contact time, flight time, and average time during the stride, as well as a greater angle progression. Correlation analyses corroborated the worsened outcomes with increasing age. This suggests that, despite being physically active, age brings about physical and physiological changes that primarily affect balance and neuromuscular capacity, impairing gait performance [12,13,20,21,22]. Additionally, the portable PodoSmart system has been used in this study, observing gait values in young and older adult populations similar to those obtained by other more sophisticated devices [23,24]. This emphasizes the usability of this wearable system to easily and comfortably obtain gait parameters in geriatric populations

Wearable systems, which are portable devices designed to monitor or record health indicators, have gained significant traction in recent years because of their versatility in monitoring physical activity and their potential to be routinely used for health and rehabilitation purposes [25]. In particular, these systems offer notable advantages, such as ease of use, portability, and efficient data processing. For example, PodoSmart is a system with technological features that make it applicable not only in laboratory settings but also in clinical environments and even for home use. Moreover, previous studies have established PodoSmart’s validity by comparing it to the Vicon system [16], while also confirming its test–retest reliability [18]. Consequently, the results of these studies have been highly favorable, especially for gait analysis, highlighting that PodoSmart is a reliable, portable, and cost-effective tool for this purpose.

To further build on this foundation, expanding the system’s evidence base is essential. This includes applying it to diverse clinical populations, with studies already conducted in populations with schizophrenia [17] and amputees [26]. However, the present study is the first conducted in the geriatric population. This is the first study to use PodoSmart in a geriatric population, which helps broaden the understanding of its potential applications and paves the way for evidence-based clinical implementation in older adults.

When analyzing the obtained gait parameters, a worsened gait efficiency is observed, expressed in parameters such as stride length, cadence, time of swing and support phase, and progression angle. These worsened parameters partially align with those highlighted by the review of Herssens et al. [3], with especially similar outcomes in terms of length of stride.

However, concerning the variable of gait speed, there is more controversy, and it largely depends on the specific age range. In our study, no significant differences were found, although older adults walk faster, and there are differences between men and women. In the Herssens et al. [3] study, gait speed decreases significantly, but only in individuals over 90 years old. Differences also exist in the Hollman et al. study [27], where gait speed decreases starting at 62 years, or in the Menz et al. [20] study, which compares adults aged 22–39 with those aged 75–85, as well as in Chung’s et al. study [21], which states that gait speed changes starting at 75 years. On the other hand, Espy et al. and Cerdá et al. argue that to compensate for the physical changes that occur with aging, older adults often adjust their gait pattern by reducing speed and shortening step and stride length, thus creating a more stable gait [28,29].

Regarding cadence, there is more variability in the results. In our study, cadence increases, as seen in the Menz et al. study [20], when comparing it on smooth surfaces, which is not the case in other studies, where no differences are found [21], or where a decrease is observed [3,12].

These spatiotemporal differences are consistent with the findings reported in the review by Klotzbier et al. [30], which analyzed interrater reliability for gait measurements in young and older adults. The review showed that data provided by PodoSmart fall within the range of differences considered reliable. Considering these findings, along with other evidence on gait speed and stride length [31]—which appear to be the most reliable parameters—the observed differences in our study exceed the minimal detectable change calculated from the standard error of measurement, indicating that they likely represent true changes rather than measurement error. Although it can be speculated that these changes in gait performance may influence functional capacity and fall risk, the lack of normative clinical reference values specific to the characteristics of the analyzed sample prevents definitive conclusions regarding their clinical significance.

While interpreting and contextualizing the results obtained, this study presents findings that corroborate the kinematic changes occurring during aging, which reflect the decline in muscle mass, the loss of mobility, and the slowing of the neuromuscular system as natural consequences of this process. These results reinforce the existing knowledge on kinematic deterioration associated with aging, although in this case, they were measured using a portable smart system, for which no data had been previously reported. Therefore, with the aim of assessing the functional capacity of older adults and, based on this, establishing therapeutic goals and monitoring their progress, clinicians may use PodoSmart as a functional assessment tool. Furthermore, this study provides reference values specifically for this population. However, special considerations should be made regarding the physical activity of the participants. In most of the cited studies, either participants’ physical activity levels were not specified, or subjects had musculoskeletal conditions affecting physical function, such as sarcopenia [32]. In contrast, in our study, all of the old adults performed 3 weekly sessions of physical activity. This could partly explain the different results observed between our study and those evaluating the general older adult population, as regular physical activity can mitigate musculoskeletal decline associated with aging and, therefore, help maintain adequate gait parameters. Notably, this may be the case for gait speed, as the comparable results of older adults to young adults could be attributed to the effect of physical activity, since this parameter has been reported to improve with regular physical activity in older adults [32]. While the present findings may offer valuable insights into the effects of physical activity on gait patterns assessed with wearable smart insoles, these results should be interpreted with caution. The absence of a comparison group of similarly aged individuals who do not engage in regular physical activity limits the ability to attribute the observed effects solely to the intervention. Therefore, future studies should include such a control group of old adults who are physically inactive to enhance the validity and generalizability of the findings. This may help to better understand how physical activity in older adults can modify gait parameters and, consequently, reflect an improved functional capacity.

PodoSmart joins a growing list of studies that have evaluated gait patterns using wearable systems. For instance, Rampp et al. [22] conducted a study where they measured stride length with the GAITRite system, which is a portable, computerized walkway used to assess spatial and temporal gait parameters by detecting footfalls and timing them. Similarly, Wipperman et al. [33] developed a digital insole that collected and analyzed force plate data using a machine learning system. This system was tested on participants with knee osteoarthritis and proved to be as reliable as traditional lab-based procedures for identifying individual-specific gait characteristics. Likewise, Arumugaraja et al. [34] designed and developed a foot-worn piezoresistive sensor system with 102 sensors to detect foot pressure distribution. By applying a hybrid filter to enhance the image quality and using machine learning models, they achieved 99.4% accuracy in detecting gait alterations related to knee pain. Hence, considering this background, we can see that increasingly, new measurement systems based on intelligent data processing models offer convenient and portable solutions for gait analysis.

### Strengths and Limitations

This study provides new evidence, as this is the first using the PodoSmart system across different age groups, detecting differences especially in terms of stride length, double support time or cadence, in line with previous studies performed with traditional lab-setting procedures. Nevertheless, some limitations should be acknowledged.

First, although significant differences were observed, the relatively small sample size limits the ability to determine whether these differences are clinically meaningful. Consequently, their implications for functional capacity or fall risk, both critical considerations in gait evaluation among older adults, remain unclear. Future research should address these aspects to better clarify the practical significance of such findings.

Second, while PodoSmart is an innovative system that has undergone reliability testing, its validity is not consistent across all gait parameters. In this study, only those variables with good-to-excellent ICC were analyzed, while measures with moderate or poor validity were excluded. This methodological decision strengthens the robustness and consistency of the results but narrows the scope of gait characteristics assessed. Further studies are needed to evaluate the measurement properties of PodoSmart and to expand the evidence base supporting the validity of its outcomes.

Third, the study did not include a control group of older adults who were not physically active, which would have allowed direct comparisons regarding the influence of regular physical activity. This limitation highlights the need for future research to explore the role of activity level in gait outcomes among older individuals.

Finally, the study may have been underpowered to detect clinically meaningful differences in subgroup analyses, particularly by sex. Future investigations should ensure sufficient sample sizes to explore sex-specific patterns in gait performance.

## 5. Conclusions

Evident differences in gait parameters were observed between young adults and active older adults using the novel wearable system, PodoSmart Insole^®^. These results are consistent with the trends reported in previous studies that employed more sophisticated laboratory-based protocols for gait analysis. Slight discrepancies between our study and earlier work may be attributed to the regular physical activity performed by the older participants, an aspect that should be explored in greater detail in future research. Furthermore, these findings expand the evidence supporting the clinical use of PodoSmart by providing data specifically for the geriatric population, which had not yet been documented with this system.

## Figures and Tables

**Table 1:** Measurements for gait analysis.

Name of Variable	Definition	ICC Value Reported
Stride length	Distance travelled by the Right foot in a gait cycle	0.912
Cadence	Number of steps taken per minute.	0.990
Speed	The speed at which the body moves in a straight line while walking.	0.916
Contact time	Duration the foot remains in contact with the ground during each step.	0.989
Swing time	The duration between foot lift and heel strike for each foot during the stride cycle is used to determine the swing time, which is the length of the section of the gait cycle when the limb is not in contact with the ground.	0.918
Double support	This is the percentage of the gait cycle during which both limbs contact the ground.	0.784
Support phase duration	The stance phase’s first sub-component. When the heel of the right foot strikes the ground and absorbs the impact, the support phase begins. It comes to an end when the toes touch the ground.	0.862
Heel strike angle	Angle at which the heel first contacts the ground during walking	0.930
Toe-off progression angle	Angle at which the toe lifts off ground at the end of the stance phase	0.975
Propulsion phase duration	The stance phase’s third sub-component. The time between the heel and toe lifting events is referred to as propulsion.	0.799
Step progression angle	During the flat foot phase, the angle between the walking path and the foot’s orientation.	0.755
Stepping	During initial contact, the angle of flexion/extension of the foot on the earth’s surface.	0.914
Circumduction	Perpendicular to the progression line, the distance between the center of each foot’s heels.	0.949

Note: The ICC values were extracted from [16,18]. ICC values are catalogued as minor reliability < 0.5; moderate reliability 0.51 to 0.75; high reliability 0.76 to 0.9; excellent reliability > 0.9.

**Table 2:** Anthropometric characteristics of the study population (*n* = 81).

	Young Adults’ Group (*n* = 40) Mean (SD)	Active Old Adults’ Group (*n* = 41) Mean (SD)	Between-Group Differences *p*-Value
Sex (male/female)	20/20	10/31	
Age (years)	26.2 (7.2)	70.1 (4.7)	<0.001 *
Height (cm)	170.5 (10.9)	158.3 (6.8)	0.200
Weight (kg)	74.1 (19.5)	69.6 (9.9)	<0.001 *
Body mass index (kg/m^2^)	25.2 (5.4)	27.8 (3.5)	0.013 *
FPI-R	3.7 (2.7)	2.0 (3.3)	0.010 *
FPI-L	3.9 (2.5)	2.4 (3.6)	0.030

Note: FPI-R: Foot Posture Index right foot; FPI-L: Foot Posture Index left foot. * indicates significant differences (<0.05).

**Table 3:** *t*-test comparison of gait parameters between young and physically active old adults.

	Foot	Young Adults (*n* = 40)	Active Old Adults (*n* = 41)	Between-Group Comparison	Effect Size
		Mean (SD)	Mean (SD)	*p*-Value	*d Cohen’s*
Stride length (m)	R	1.39 (0.10)	1.33 (0.10)	0.009 *	**0.59**
	L	1.40 (0.10)	1.33 (0.11)	0.011 *	**0.66**
Cadence (steps/minute)	Both	112.7 (8.4)	123.12 (5.63)	0.000 *	**1.46**
Speed (m/s)	Both	4.74 (0.47)	4.90 (0.35)	0.097	0.38
Contact time (ms)	R	639.19 (54.50)	594.50 (37.55)	0.000 *	**0.96**
	L	634.84 (53.10)	583.292 (46.07)	0.000 *	**1.04**
Swing time (ms)	R	408.18 (27.01)	365.707 (18.06)	0.000 *	**1.85**
	L	408.02 (25.57)	366.707 (19.65)	0.000 *	**1.81**
Double support time (ms)	R	114.90 (20.76)	107.16 (14.14)	0.054	0.44
	L	114.00 (20.84)	106.93 (14.10)	0.079	0.40
	Mean	114.45 (20.73)	107.04 (14.12)	0.065	0.42
Support phase time (ms)	R	101.70 (13.12)	87.70 (10.92)	0.000 *	**1.16**
	L	103.15 (12,99)	89.12 (17.10)	0.000 *	**0.92**
Heel strike angle (deg)	R	−20.09 (4.25)	−18.8 (4.74)	0.204	0.28
	L	−17.49 (4.15)	−16.24 (4.29)	0.187	0.29
Toe-off progression angle (deg)	R	−2.717 (4.30)	−0.975 (3.19)	0.042 *	**0.46**
	L	−5.49 (4.28)	−3.41 (2.91)	0.012 *	**0.57**
Propulsion time	R	223.87 (38.52)	233.41 (19.49)	0.163	0.30
	L	1.49 (0.64)	1.36 (0.45)	0.264	0.23
Step progression angle (deg)	R	11.17 (5.77)	11.58 (4.41)	0.721	0.08
	L	6.71 (5.09)	8.51 (4.54)	0.012 *	**0.37**
Stepping (deg)	R	25.45 (3.41)	23.43 (6.31)	0.078	0.40
	L	27.66 (2.97)	26.43 (5.65)	0.227	0.27
Toe off angle (deg)	R	53.56 (8.37)	53.63 (4.58)	0.961	0.01
	L	52.47 (8.65)	52.68 (5.30)	0.895	0.03
Circumduction (cm)	R	3.12 (1.44)	3.04 (0.83)	0.774	0.07
	L	2.81 (1.29)	2.46 (0.77)	0.142	0.33

Note: R: Right foot; L: left foot; m: m; m/s: m/s; ms: milliseconds; deg: degrees. Significant values (*p* < 0.05) are marked in bold and with *.

**Table 4:** Comparison of gait parameters per sex and group. Men in young adults: *n* = 20; women in young adults: *n* = 20; men in active old adults: *n* = 10; women in active old adults: *n* = 31.

Measure	Foot	Sex	Young Adults		Active Old Adults		Between-Groups
			Mean (SD)	*p*-Value	Mean (SD)	*p*-Value	*p*-Value
Stride Length (m)	R	Men	1.41 (0.09)	0.403	1.40 (0.03)	0.019 *	0.792
		Women	1.38 (0.11)		1.31 (0.09)		0.021 *
	L	Men	1.41 (0.08)	0.295	1.39 (0.01)	0.059	0.607
		Women	1.38 (0.11)		1.31 (0.11)		0.049 *
Cadence (steps/min)	Both	Men	109.76 (7.99)	0.026 *	120.10 (3.60)	0.050 *	0.001 *
		Women	115.64 (8.02)		124.09 (5.86)		<0.001 *
Speed (m/s)	Both	Men	4.65 (0.43)	0.207	5.03 (0.42)	0.221	0.033 *
		Women	4.48 (0.51)		4.87 (0.32)		0.866
Contact time (ms)	R	Men	663.99 (54.89)	0.003 *	597.20 (38.42)	0.128	0.002 *
		Women	614.40 (42.29)		576.32 (36.43)		0.001 *
	L	Men	658.99 (55.06)	0.003 *	595.60 (37.90)	0.338	0.003 *
		Women	610.70 (39.10)		579.32 (48.30)		0.019 *
Swing time (ms)	R	Men	417.05 (31.30)	0.036 *	378.20 (13.87)	0.010 *	0.001 *
		Women	399.31 (18.77)		361.67 (17.56)		0.000 *
	L	Men	415.97 (29.34)	0.048 *	379.40 (17.92)	0.017 *	0.001 *
		Women	400.07 (18.67)		362.61 (18.65)		0.000 *
Double support time (ms)	R	Men	123.25 (20.15)	0.009 *	109.27 (16.33)	0.594	0.068
		Women	106.55(18.21)		106.48 (13.58)		0.987
	L	Men	122.88 (20.22)	0.005 *	108.98 (16.21	0.604	0.070
		Women	105.12 (17.81)		106.27 (13.58)		0.796
	M	Men	123.07 (20.18)	0.007 *	109.12 (16.27)	0.599	0.069
		Women	105.84 (17.84)		106.37 (13.58)		0.903
Support phase time (ms)	R	Men	99.44 (14.38)	0.282	84.20 (5.41)	0.102	0.003 *
		Women	103.96 (11.65)		88.83 (12.04)		<0.001 *
	L	Men	100.64 (12.93)	0.226	90.30 (9.95)	0.806	0.035 *
		Women	105.67 (12.89)		88.74 (18.96)		<0.001 *
Heel strike angle (deg)	R	Men	20.96 (4.87)	0.200	23.00(3.97)	0.001 *	0.263
		Women	−19.22 (3.43)		−17.45 (4.18)		0.122
	L	Men	18.66 (4.32)	0.076	19.70 (4.90)	0.002 *	0.557
		Women	−16.33 (3.72)		−15.12 (3.48)		0.248
Toe-off angle (deg)	R	Men	−1.75 (4.45)	0.159	−0.60 (1.71)	0.675	0.039 *
		Women	−3.68 (4.03)		−1.09 (3.56)		0.020 *
	L	Men	−4.98 (3.90)	0.455	−2.60 (2.54)	0.316	0.093
		Women	−4.98 (3.90)		2.60 (2.54)		**0.057**
Step progression angle (deg)	R	Men	13.54 (6.37)	0.008 *	14.50 (3.53)	0.014 *	0.665
		Women	8.80 (3.98)		10.64 (4.30)		0.131
	L	Men	8.457(5.79)	0.030 *	10.30 (6.10)	0.155	0.417
		Women	4.98 (3.65)		7.935 (3.85)		0.009 *
Stepping (deg)	R	Men	25.09 (3.74)	0.513	26.90 (4.58)	0.045 *	0.258
		Women	25.81 (3.10)		22.32 (6.45)		0.029 *
	L	Men	27.97 (3.30)	0.521	29.70 (5.18)	0.034 *	0.275
		Women	27.36 (2.65)		25.38 (5.49)		0.092
Toe off angle (deg)	R	Men	52.58 (7.83)	0.469	53.70 (4.92)	0.959	0.686
		Women	54.53 (8.97)		53.61 (4.55)		0.674
	L	Men	51.21 (8.49)	0.366	51.70 (7.14)	0.600	0.879
		Women	53.72 (8.84)		53.00 (4.66)		0.737
Circumduction (cm)	R	Men	3.34 (1.45)	0.339	3.20 (0.63)	0.517	0.768
		Women	2.90 (1.42)		3.00 (0.89)		0.766
	L	Men	3.03 (1.31)	0.299	2.80 (0.63)	0.117	0.605
		Women	2.59(1.27)		2.35 (0.97)		0.404

Note: Comparison of variables differentiating participants of the opposite sex in the same group of subjects (men vs. women, same age range). Comparison of variables differentiating participants of the same sex in different subject groups (young vs. old, same sex). R: Right foot; L: left foot; m: m; m/s: m/s; ms: milliseconds; deg: degrees. Significant differences (*p* < 0.05) are marked in bold and with *.

**Table 5:** Pearson correlation analysis between gait parameters and age (*n* = 81).

Independent Measure	Mean (SD)	Dependent Measure	Mean (SD)	*Pearson r*	*p*-Value
Age (years)	48.15 (6.12)	Right stride length (m)	1.36 (0.10)	−0.285	0.010 *
		Left stride length (m)	1.36 (0.11)	−0.287	0.009 *
		Cadence (steps per minute)	117.97 (8.84)	0.590	<0.001 *
		Speed	4.83 (0.43)	0.176	0.117
		Right Contact time (ms)	609.94 (54.75)	−0.504	<0.001 *
		Left Contact time (ms)	608.75 (55.76)	−0.462	<0.001 *
		Right Swing time (ms)	386.68 (31.23)	−0.662	<0.001 *
		Left Swing time (ms)	387.1 (30.73)	−0.66	<0.001 *
		Right Double support (ms)	110.98 (18.04)	−0.192	0.087
		Left Double support (ms)	110.42 (18.00)	−0.172	0.124
		Mean Double support (ms)	110.7 (17.98)	−0.182	0.103
		Right duration of the support phase (ms)	94.61 (13.90)	−0.524	<0.001 *
		Left duration of the support phase (ms)	96.05 (16.69)	0.439	<0.001 *
		Right foot propulsion phase (ms)	226.92 (31.32)	0.117	0.299
		Left foot propulsion phase (ms)	228.7 (30.60)	0.173	0.122
		Right Step progression angle (deg)	11.38 (5.10)	0.049	0.663
		Left Step progression angle (deg)	7.63 (4.88)	0.185	0.099
		Right Stepping (deg)	24.44 (5.16)	−0.224	0.045
		Left Stepping (deg)	27.05 (4.55)	−0.115	0.227
		Right Toe off angle (deg)	53.60 (6.68)	−0.002	0.141
		Left Toe off angle (deg)	52.58 (7.11)	0.001	0.986
		Right Circumduction (cm)	3.08 (1.17)	−0.007	0.951
		Left Circumduction (cm)	2.63 (1.08)	−0.146	0.193

Note: m: m; m/s: m/s; ms: milliseconds; deg: degrees. Significant differences (*p* < 0.05) are marked in bold and with *.

## Data Availability

Data will be available under reasonable request to corresponding author.
